# Knowledge, attitudes and practices on cervical cancer screening among the medical workers of Mulago Hospital, Uganda

**DOI:** 10.1186/1472-6920-6-13

**Published:** 2006-03-01

**Authors:** Twaha Mutyaba, Francis A Mmiro, Elisabete Weiderpass

**Affiliations:** 1 Department of Obstetrics and Gynaecology, Mulago Hospital, Makerere Medical School, P.O.Box 7051, Kampala, Uganda; 2 Department of Medical Epidemiology and Biostatistics, Karolinska Institutet, Stockholm, Sweden; 3 The Cancer Registry of Norway, Montebello N-0310, Oslo, Norway

## Abstract

**Background:**

Cervical cancer is the commonest cancer of women in Uganda. Over 80% of women diagnosed in Mulago national referral and teaching hospital, the biggest hospital in Uganda, have advanced disease. Pap smear screening, on opportunistic rather than systematic basis, is offered free in the gynaecological outpatients clinic and the postnatal/family planning clinics. Medical students in the third and final clerkships are expected to learn the techniques of screening. Objective**s **of this study were to describe knowledge on cervical cancer, attitudes and practices towards cervical cancer screening among the medical workers of Mulago hospital.

**Methods:**

In a descriptive cross-sectional study, a weighted sample of 310 medical workers including nurses, doctors and final year medical students were interviewed using a self-administered questionnaire. We measured knowledge about cervical cancer: (risk factors, eligibility for screening and screening techniques), attitudes towards cervical cancer screening and practices regarding screening.

**Results:**

Response rate was 92% (285). Of these, 93% considered cancer of the cervix a public health problem and knowledge about Pap smear was 83% among respondents. Less than 40% knew risk factors for cervical cancer, eligibility for and screening interval. Of the female respondents, 65% didn't feel susceptible to cervical cancer and 81% had never been screened. Of the male respondents, only 26% had partners who had ever been screened. Only 14% of the final year medical students felt skilled enough to use a vaginal speculum and 87% had never performed a pap smear.

**Conclusion:**

Despite knowledge of the gravity of cervical cancer and prevention by screening using a Pap smear, attitudes and practices towards screening were negative. The medical workers who should be responsible for opportunistic screening of women they care for are not keen on getting screened themselves. There is need to explain/understand the cause of these attitudes and practices and identify possible interventions to change them. Medical students leave medical school without adequate skills to be able to effectively screen women for cervical cancer wherever they go to practice. Medical students and nurses training curricula needs review to incorporate practical skills on cervical cancer screening.

## Background

Cervical cancer is the commonest malignancy of women in Uganda [1,2]. Over 80% of patients diagnosed with cervical cancer in Mulago hospital present with advanced disease, [3,4]. Cervical cancer is largely preventable by effective screening programmes and considerable reduction in cervical cancer incidence and deaths has been achieved in developed nations with systematic cytological smear screening programmes [5-7]. These have not been possible in most low resource settings including Uganda. The only available activity has been to use opportunistic screening of those women who come to the health units for other reasons. Screening the women then becomes the responsibility of the medical worker who should know those eligible [8]. It is known that precancerous lesions are detectable for 10 years or more before cancer develops. The ideal ages for screening should then be 30–40 years, the age when women are at the highest risk of precancerous lesions [9]. However, younger women who have been sexually active should be screened as they might have lesions and even cancer especially if they have HIV infection [10-13]. Studies have shown that primary health nurses can be trained to screen women for cervical cancer [14-16].

No previous study has assessed the knowledge and practices of medical workers on prevention of cervical cancer in Mulago, the national referral and teaching hospital for Makerere University, where most cervical cancer patients are referred for treatment. This hospital is the major training institution for medical workers in Uganda, and therefore it should be the trendsetter for best practice regarding cervical cancer prevention and management. What is known and done in this teaching institution gives an insight on what the rest of the medical workers elsewhere in the country, and more broadly, in the East African region, know and do.

## Methods

We conducted a cross sectional survey at Mulago hospital, situated in Kampala, Uganda. This is the largest hospital in Uganda and is the national referral hospital as well as teaching hospital for Makerere Medical School – the most prominent University in the region. We obtained a complete list of the hospital medical workers who make primary clinical contact with women eligible for cervical cancer screening such as specialists, medical officers, final year medical students, and nurses. Medical workers that were presumed not to come clinically in contact with female patients, e.g. laboratory workers, were disregarded.

We interviewed a sample of 310 medical workers weighted according to the representation of each category to the total work force. Participants answered a self-administered questionnaire containing both coded and open-ended questions about knowledge, attitudes and practices regarding cervical cancer screening. The interviews took place between November and December 1998.

We considered knowledge about risk of cervical cancer good if a respondent mentioned at least 3 of the known risk factors (early sexual debut, multiple sexual partners, multiparity, low social economic status, Human Papilloma Virus infection). We elicited knowledge about eligibility for screening and screening interval according to WHO guidelines. Practice was evaluated as screening patients for cervical cancer, referring patients for screening, and in the case of female responder, having ever been screened themselves. Attitudes referred to the various reasons for not screening patients, not referring patients for screening, and not getting screened themselves.

The data was processed using the EPI-INFO programme. All participants gave informed consent. The study protocol was approved by the local ethical committee, Mulago Hospital.

## Results

Of the invited 310 medical workers, 288 (93%) accepted to participate in the study. They were specialists (19), medical officers (39), final year medical students (63), and nurses/midwives (167). Three of the questionnaires were incomplete and were excluded from the analysis leaving 285. Most of the participants were female (69%) and 90% had ever had sexual intercourse themselves.

Most participants knew that the cancer was curable if detected at an early stage, and that Pap smear screening could detect early cervical lesions. However, knowledge of risk factors for cervical cancer and details of screening activities was very low. (Table 1)

Among the final year medical students, 87% had never done a pap smear, 56% had never done a speculum examination and only 14% felt skilled enough to use speculum.

Most of the study participants routinely managed female patients, and had frequently performed vaginal examinations, although use of speculum was surprisingly low (12%). Only 19% of the female medical workers had ever had a cervical cancer screening test done, and the vast majority never asked patients if they had been screened for cervical cancer or ever referred patients for screening (78%). (Table 2)

## Attitudes

Of all respondents, 93% thought cancer of the cervix was a public health concern, 68% thought that it was easy to diagnose and 65% of the participant females did not think they were susceptible to cervical cancer themselves, while 60% of males thought that their partners were susceptible. Most nurses and midwives thought that speculum examination and Pap smear were doctors' procedures, while 22% of the medical students thought they were for senior doctors only. Doctors in disciplines other than gynaecology thought that speculum examination was an activity for gynaecologists only. Lack of vaginal specula and absence of indication for speculum examination were common reasons for not screening patients.

Among the females respondents, reasons for not having been screened included: not feeling at risk, lack of symptoms, carelessness, fear of vaginal examination, lack of interest, test being unpleasant and not yet being of risky age. Moreover, 25% of the female respondents said that they would only accept a vaginal examination by a female health worker. Medical students were asked for strongest reason for not taking Pap smears. Responses were: 35% thought they were not allowed, 15% never thought about it, 22% thought it was for senior doctors and 26% did not know how to do one.

## Discussion

The majority of respondents in our survey correctly identified cervical cancer as a major public health problem. This was expected as cervical cancer patients represent an important proportion of the gynaecological wards occupants.

Despite being the commonest cancer of women in Uganda, there is no systematic screening programme for cervical cancer. Pap smear based screening programmes are not feasible in low resource settings like Uganda due to economical and logistic reasons like lack of trained pathologists, and equipped laboratories. Visual inspection is an alternative method that was tested and promises to be more feasible for low resource settings like Uganda [17,18].

In the absence of a systematic screening programme the expected practice is to opportunistically screen eligible women when they come to health units for other reproductive services. In the opportunistic screening system, the onus is on the health worker who handles the eligible women to offer screening or refer her to a unit where screening could be done. In Mulago hospital Pap smear is exclusively performed in the gynaecological department and women from other units should be referred to the gynaecological unit for screening. This study identified lack of a clear policy on who should do the screening. Studies have shown it is possible to train midwives/nurses to screen for cervical [14-16]. Attitudes that it is for doctors or gynaecologists need to change.

The majority of respondents in our study were nurses, who form the bulk of medical workers in most health units in Africa. In departments other than Gynaecology, the negative practice of not screening the patients who came under their care could be attributed to their routines, but this would not explain the reluctance to get screened themselves despite the availability of a free service almost any time they wished to. Most agreed that it was a public health problem, they knew about the Pap smear test, and that cervical cancer is curable if detected early. Despite that, 81% eligible female respondents had never been screened, mostly because they did not feel vulnerable to the disease. It is unlikely that these medical workers would feel motivated to screen others or advise them accordingly. Similar attitudes have been reported among medical workers regarding HIV testing.

Some of the reasons given like lack of specula were valid as many of the medical workers did not work in the department of gynaecology. Referral for screening in the gynaecology department was also very low, reflecting an absence of policy on health promotion and disease prevention in Mulago hospital, which is the national referral and teaching hospital and the trendsetter for best practice in the country. The students trained in this institution will practice what is taught and practised in the training institution. Only 14% felt skilled enough to use a vaginal speculum. The findings among the medical students were similar to a study in Australia [19]. Medical training in Australia is similar to that in Uganda (British) and these results indicate a weakness in the curriculum, which needs to be corrected.

## Conclusion

Overall, the study results indicated a big gap regarding cervical cancer screening in Mulago, the national referral and teaching hospital in Uganda. This was at variance to the reality of the public health importance of cervical cancer in Uganda. Indeed a situational analysis done in 5 countries in east, central and southern Africa found minimal screening activities despite available facilities for Pap smear in 95% of sampled health units [20].

## Recommendations

There is need for sensitisation of health workers about cervical cancer and importance of screening. Training curricula of nurses and medical students need to be revised to include more practical cervical cancer screening skills. There is need to change attitudes that screening is only for gynaecologists. For opportunistic screening to work, health workers in other departments need to be sensitised on the gravity of cervical cancer and to remember to refer all eligible women who come into their care for screening.

The findings in this study are descriptive. Whereas one can attribute the failure to screen clients to gaps in training curriculum and lack of clear policy guidelines on screening for cervical cancer, further research is needed to explain the reticence to get screened by eligible health workers despite knowledge about the problem and ready access to screening facilities in the gynaecological department. Health providers need to be targeted first because of their pivotal role in any planned future screening programmes.

## Competing interests

None declared by any of the authors. The funding agency had no influence in the design, execution or interpretation of the results presented here.

## Authors' contributions

T. Mutyaba and F.A. Mmiro conceptualised the study. T Mutyaba was responsible for the fieldwork, data management and analysis of the data, and wrote the study report. E. Weiderpass motivated Twaha to publish the study as an article, helped with the interpretation of the results and writing of the manuscript.

## Pre-publication history

The pre-publication history for this paper can be accessed here:



## Figures and Tables

**Table 1:** Knowledge about cervical cancer and screening

**Knowledge**	**Percentage with good knowledge**
Whether cervical cancer is curable	81%
Risk factors for cervical cancer	29%
Of pap smear screening	83%
Eligibility for screening	26%
Screening interval	39%

**Table 2:** Practices on cervical cancer screening

**Practices**	**Percentage**
Routine management of female patients	86%
Frequently performing vaginal examinations	62%
Speculum use during vaginal examinations	12%
Females respondents who have ever been screened	19%
Males whose partners have ever been screened	26%
Don't ask patients whether screened	78%
Don't refer patients for screening	78%
